# Honor Killings in the Eastern Mediterranean Region: A Narrative Review

**DOI:** 10.3390/healthcare11010074

**Published:** 2022-12-27

**Authors:** Sarah M. AlQahtani, Danah S. Almutairi, Eman A. BinAqeel, Reema A. Almutairi, Reem D. Al-Qahtani, Ritesh G. Menezes

**Affiliations:** College of Medicine, Imam Abdulrahman Bin Faisal University, Dammam 34212, Saudi Arabia

**Keywords:** Eastern Mediterranean region, honor killing, violence against women, femicide, homicide

## Abstract

Honor killing is a violent crime committed by one or more perpetrators, in which the crime’s intention is to restore honor to their family. In this narrative review, the authors investigate the epidemiology of honor killing in the Eastern Mediterranean Region. Furthermore, the social, cultural, and legal aspects of honor killing are discussed. Numerous socio-cultural factors lead to the action of killing for honor in this region. They include deeply rooted patriarchal dominance, the desire to maintain social status, and being poorly educated. Honor killing perpetrators have similar characteristics, such as rating female chastity at a higher price and justifying violence against women. The impact of honor killing on family members is much greater than the perceived families’ beliefs of the community’s rejection of the female’s dishonorable behavior. Silence culture dominates these societies, and many crimes are under-reported in this region. Often, a judicial trial is not conducted for such heinous crimes. Penal code reforms, campaigns to promote human rights, steps to improve the education level, and active participation of civil society in condemning such crimes are a few essential measures that need to be considered in order to curb the social evil of honor killing.

## 1. Introduction

Honor-based violence (HBV) can be defined as any form of violence or abuse that is conducted to protect the honor of a family or community [1]. Victims of honor crimes are most often females. Boys and men can also be victims; however, it is much less common [1]. 

Being illiterate, unemployed, economically vulnerable, having inadequate social support, and lacking knowledge regarding legal rights are factors that can impose a greater risk of becoming a victim of violence [2]. Furthermore, the patriarchal society, which recognizes the man to be the head of the family who is in charge of fighting against any dishonoring act, places a high psychological burden on females living in these societies, and in some regions, this patriarchal system goes as far as considering women to be men’s property [2,3]. 

Behaviors that might subject females to punishments include getting pregnant or giving birth outside the range of marriage, being a victim of rape or sexual assault, or refusing an arranged marriage. Moreover, if a female wishes to divorce due to abuse or any other cause, marry a person of her own choice, or even attend university or college against the guardian’s will, she might be subjected to violence. This sometimes includes the simple act of talking to men in any context, even if out of necessity [1,4]. 

HBV can range from physical assault, domestic abuse, sexual harassment or violence, forced abortion, forced marriage to their rapist, threats of killing, and being poisoned to the most extreme form of HBV, which is honor-based killing. Honor killing (HK) is considered a violent crime committed by one or more perpetrators, usually a male family member, against a woman who committed alleged misbehaviors or acts that brought shame to the family, which the crime is supposed to erase, restoring honor to their family [5]. 

According to the World Health Organization (WHO) in 2012, it was estimated that around 5000 murders occur each year worldwide in the name of honor. Studies have reported that commonly used methods in honor crimes include strangulation, stabbing, burning, acid burning, stone throwing, being buried alive, and forcing a female to throw herself from a window or forcing her to take poison [1,6]. Sometimes, weapons are used, such as axes, firearms, and edged tools [6]. 

The way honor is dominantly conceptualized in many “honor-based” communities, notably in the Eastern Mediterranean Region (EMR), has led to many crimes being committed in the name of honor. These cultures place significance on women’s general social and sexual behavior, and the value of women’s behavior is what explains the HK phenomena in these communities. The family and social network are worshipped in these cultures; as a result, HK is a collective act that is decided by a family or a group council. It is driven by certain cultural norms regarding honorable acts rather than by individual choices or desires. Honor killings eventually need group approval for confirmation and validity. The HK rate in the countries of the EMR is variable but consistently alarmingly high. Therefore, in this paper, the authors intend to shed light on honor killing in the EMR and its legal implications, as well as discuss the socio-cultural aspects surrounding this particular region and elaborate on the factors that could possibly be contributing to the high prevalence reported in the EMR [6,7,8]. In addition, this paper addresses the epidemiology of honor crimes in the countries included in this region and the attitudes of the citizens of those countries towards HK. 

## 2. Epidemiology

### 2.1. Gulf Cooperation Council (GCC) Countries

There are not many studies on HK reported in Saudi Arabia. However, researchers found that Saudi Arabia has an approximately 58% rate of domestic violence, which, in comparison to the worldwide rate, which lies between 10–52%, is significantly higher. Note that this rate only encompasses the main cities in Saudi Arabia, as the study in question did not include rural areas [9].

However, while stories rarely come out in legitimate news outlets in most cases, nowadays, more HK stories make it to the surface with the help of modern-day social media. These platforms are perceived as unreliable, forming what is often referred to as gray literature (e.g., newspaper articles, social media posts, etc.).

Similar socio-cultural factors are shared between Saudi Arabia and the other Gulf Cooperation Council (GCC) countries. Therefore, many cases are also under-reported and thus cannot be epidemiologically estimated.

### 2.2. Other Countries in the Eastern Mediterranean Region

Some tragic incidents are not officially reported and often go around as “peer-say” and “tales”, making it difficult to pin down the truth and report it. Additionally, this lack of incidents being reported is due to socio-cultural factors that justify honor killings. Furthermore, relatives and other members of society will help cover it up if need be. Cover-up stories are various, for example, firearm-cleaning accidents. Such alleged “accidents” are supported by males of the same society and thus end up conveyed in the media as such; therefore, honor killing is now non-existent and unreported.

In Jordan, 50 HKs were reported between the years 2000 and 2010 [7]. In 1997, 52 cases took place in Egypt, and in Yemen, 400 women were victims of HK in the same year. In Lebanon, 38 cases of HK were reported between 1996 and 1998 [7]. In Palestine, 27 cases in 2014 and 15 cases in 2015 were reported by the Women’s Center for Legal Aid and Counselling. However, these small numbers can be an underestimation of the problem [10,11]. 

In the Libyan civil war, according to campaigners for the US-based organization Physicians for Human Rights, there was a case of three adolescent girls who were raped by the ruling forces at a school in Misrata Town. Later, their father killed them for dishonoring the family [12]. 

The highest number of HKs is believed to be in Pakistan. A total of 4101 cases of honor crimes have been reported to the court in the period between 1998 and 2003 by the Pakistan Ministry of Interior. Furthermore, the Human Rights Commission of Pakistan reports a steady increase in the frequency of honor killings; in 2013, 869 cases of HK were reported, while in 2014, it was estimated as 1000 cases, and in 2015, there were 1100 cases [7]. Additionally, a study in Pakistan published in the year 2009 aimed to put HKs in numbers; it estimated that during the time of the study (4 years), 1957 women were killed after allegations of extramarital affairs. A total of 88% of those women were married. Furthermore, 18% of women killed were under the age of adulthood (<18 years old) [13]. 

In Afghanistan, 243 cases of HK were registered, according to the Afghanistan Independent Human Rights Commission, between March 2011 and April 2013. Of those cases, only 56% of perpetrators were identified. The majority (39%) were husbands, 15% were brothers, 9% were fathers, 6% were brothers-in-law, 5% were other family members, and the remaining 26% were relatives [11]. 

In Iran, there were HKs in less developed areas, such as Khuzestan and Kurdistan. The Ministry of Justice in Khuzestan reported 54 HK cases in 2004. More than 40% of murders occurred in the name of honor, as reported by the police department in Khuzestan [7]. Another study was conducted in Iran. There were about 8000 reported HKs between the years 2010 and 2014, specifically in some regions such as East Azerbaijan province, where the estimated reports of all murders have reached 20%, and 50% of family murders are related to honor issues. It was found that in regions with increased rates of HKs, there is a parallel increasing rate of unemployment and poverty. Moreover, during the COVID-19 era, incidences of HKs increased not only in Iran but also worldwide. It is perceived that factors such as lockdown, isolation, and financial insecurity during the COVID-19 pandemic led to increased stress and poorer socio-economic status among individuals. This impact has a greater effect on the disadvantaged group, which might increase the tendency of men to practice violence [14]. 

## 3. Social and Cultural Aspects Contributing to Honor Killing

### 3.1. The Development of Fragile Masculinity and Its Ties to Honor Killing

“You are the man of the house” has been known over the years to be this seemingly harmless narrative pushed upon young boys to boost their masculinity early on in their lives. It was, and still is to this day, a raising technique used to direct young males to fall into the masculine roles cultivated for them by their parents (mostly the father) to step in and take charge of the household’s females, who are often of similar age to the boys or even older, to relieve the parents from the burden of raising them. 

This exact same narrative is the reason a gender striation now exists, where the male is perceived to be the “guardian” of the female and her honor when initially, all of this only started as a harmless phrase to embrace boys’ masculinity. Over time, it progressed into being an actual societal power play that females all over the world must live with to this moment [15]. 

Over time, this narrative directed boys into growing the belief that their household’s females’ “reputations” were their burdens to bear, and they must keep it untarnished at all costs. Those females were no longer their own persons but rather, an extension of those males’ masculinity and “ability to tame”. That led to the birth of the association “if you cannot keep your females in line, you are not a respectable masculine male” and the rise of the concept of “fragile masculinity”, which is when the male’s sense of masculinity is fractured upon feeling threatened by the slightest loss of control over female members of the family, even if the male is not their actual legal guardian (e.g.: father) but rather, the females’ male sibling, cousin, etc. [15].

After having now developed a basic understanding of masculinity as a social construct and how distorted and fragile its foundation is in our modern society, the following section will illustrate ways by which this fragile masculinity evolved to build a deeply patriarchal society, where a man’s worth relies heavily on his family’s feminine entities and the extent of his control over them. 

### 3.2. Demonstration of the Patriarchal Society

Because a man’s honor is merely an extension of that of his female relatives, the patriarchal society has been passing down the same agenda of guarding the female honor—represented by her sexual virginity—among males generation after generation. Even if a female’s dad passes away, she would still have her male sibling/cousin ensuring her chastity until she gets bonded to a new honor-guarding male in a family-approved marriage, as those disapproved by the family are punishable too [16,17]. In case the chastity in question is tainted in any way before the female gets married, sometimes by something as minor as a rumor, that female could endure serious consequences, ranging from being locked up and denied a social life and/or education to extreme fates, such as death [15,16]. Addressing the issue objectively, one could argue that it is the family’s right in some cultures and religions to make their females wait until marriage to have sexual intercourse. However, this begs the question: what happens if the female was raped before marriage? Sadly, the patriarchal society does not consider females and their tragedies in its views and conduct, as in such cases, the female is still perceived as shameful to the family, and the same punishments previously discussed apply [18]. 

In cases where the family members hesitate to commit the act of HK, they are subjected to consistent severe peer pressure by their surrounding society (e.g.: neighbors, relatives, etc.) and are discouraged from showing the slightest empathy towards their raped or assaulted female family members. They are instead encouraged to punish them and view them as shameful and reject them, all in an effort to eventually restore/maintain the family’s status within the society [15,18]. 

These tragedies can be attributed to the fundamental flaw in the societal system, which is forcing a large proportion of males to view females in a patriarchal light from a very young age, progressively solidifying the rightfulness of HK as they get older [15]. An example would be the case of the two sisters, Aisha and Gamila, who were born in Yemen. The two sisters were subjected to patriarchal attitudes by their father from a very young age. The fear of them becoming hyper-sexual made their father subject them to having female circumcision (female genital mutilation). As the years passed and the girls began to mature, their father’s concern about their purity also grew to the extent of isolating them and not allowing them to attend school. Following that time, their father decided it was the appropriate time for them to have an arranged marriage. Both girls then carefully planned and managed to escape outside the country. Since then, the girls are considered to have brought shame and dishonor to their family, and as said by their father, “their blood must be shed in order to cleanse the family name”. This case supports the idea that a patriarchal society can go to the extremes of permitting the rightfulness of HK [19]. 

### 3.3. Social Profile of Perpetrators 

According to a 2019 study on “factors associated with honor killing” having a low socio-economic status played a significant role in raising the rates of honor-based crimes [11,20]. 

Other factors were having a history of violence against women in the families of the victim and perpetrator and their surrounding community. Some things the study included as violence were being beaten by a sibling or a parent, childhood traumas, violence from a husband or mother-in-law, and so on. An additional factor associated with HK is being part of a society with patriarchal gender attitudes, as well as having higher levels of poverty and the need for borrowing because of hunger [11,14,20]. Regarding poverty, a Jordanian study discussed how Syrian refugees were especially prone to sexual and gender-based violence, including honor killings, as a result of the country’s declining economic conditions since the start of the war in Syria [21]. 

Moreover, another factor that was found to play a vital role in cultivating a patriarchal society is poor education, as well as being misinformed at a young age about gender roles and “family honor” and where it lies, which unfortunately was found to be taught to the younger generation, even at schools [14,15]. A study previously mentioned that academic education plays an important role as a protective factor against honor killing [11]. Additionally, there is a link between cousin marriage and HBV. HK can be used to preserve ethnic boundaries. It is a system that depends on the younger generations’ obedience to survive [22]. 

Another crucial factor worthy of mentioning that contributes to the significant increase in the prevalence of HK is rapid modernization, where the world is advancing too fast for the perpetrators’ stubborn and rigid patriarchal mindsets [20]. 

## 4. After-Effects of Honor Killing 

In the Middle East, the family is a valuable part of the culture. For instance, Palestinians view their lives as members of a family rather than as individuals. There is a lack of studies on the long-term effects of HK on families and the impacts of such a severe type of violence against women [23]. 

A Palestinian study used grounded theory to investigate the socio-emotional impacts of HK on 23 family members of murdered women. The study’s main result was that contrary to the murderers’ assumptions, killing female relatives did not restore family “honor”, nor did it improve it. Contrariwise, it damaged the families’ reputations by separating them from their communities, exacerbating their social isolation, rejection, and stigmatization [23]. 

Social death impact went beyond the individual’s emotional and social damage to the family’s ties, resulting in them being stigmatized in their own community. Many sources of stigma were mentioned, namely family, friends, neighbors, schools, and the employment market. Social death, in which people are treated as though they are dead, has caused a change in roles and status in society, as well as failed relationships. Consequently, family members felt powerless to oppose this treatment [23]. 

Similar to other Arabs, the Palestinian community has unique social support attributes, such as large community bonds and mourning rituals, in which many people give their condolences to the deceased. Sadness conveys to the community that a beloved family member has died. During the grieving phase, visitors often take care of the bereaved and relieve them from doing basic chores (e.g., preparing meals for them). Thus, social interaction and support are considered essential in the grieving process. HK victims’ families, on the other hand, were rejected, shamed, and socially isolated. Several troubles arose with the family members’ reactions to the death of their beloved one because of the community’s judgment and interactions, hence making the grieving process more complicated [23]. 

Usually, family members form two types of bonds: private and social. The private bond includes emotional connection with the victims, talking with the departed, carrying their images, silently crying, and constantly thinking of them. While the social bonds includes mourning rituals, grave visiting, wearing black clothing, hanging victims’ photos, or even keeping their belongings untouched. However, HK victims’ families experience extreme emotional distress because society or family members neither allow nor acknowledge these two types of bonds [23]. 

HK victims’ families described persistent lasting pain and a sense of futility in the face of death, along with sentiments of guilt and self-blame. Furthermore, in cases where the perpetrators were not punished, a loss of security was sensed. This occurred in the context of cultural stigma and exclusion from their close community, which also held them responsible for the murder. While the victims’ suffering came to an end with death, for those left behind amid the chaos, it was merely the beginning [23]. 

## 5. Attitudes towards Honor Killing

While many studies have investigated the social and legal context of HK, there have been few studies on the normative support for HKs and the factors that impact the attitudes toward it at an individual level. As a result, a Jordanian study aimed to analyze attitudes toward HK by performing a quantitative study with the use of a questionnaire that was distributed to 856 ninth graders in Amman, Jordan’s secondary schools. According to the findings, a sizable number of respondents thought it was acceptable to kill a female family member who had dishonored the family. HK was found to be acceptable by approximately twice as many boys as they were by girls [24]. 

Support for HK was higher among adolescents from low-education families and the traditional ones with five or more children. Furthermore, it was also supported by adolescents who held collectivist and patriarchal perspectives, valued female chastity, and ethically neutralized violent behavior in general. HK was not considered a crime, but rather, a justifiable reaction to the victim’s disgraceful defilement, a deed that restores the family’s honor, hence presenting such behavior as respectable [24]. 

Regarding parenting, HK attitudes were not predicted by the mother’s harsh discipline. However, it was predicted by harsh discipline from the father, but interestingly, the impact was shown among the boys only. It demonstrated that a father’s dictatorial and patriarchal disciplinary approach enhanced the chances that his son will believe that it is acceptable to kill a woman to protect their family’s honor. Lastly, after adjusting over several variables, the findings revealed that religion and the intensity of religious beliefs had no bearing on HK support [24]. 

In 2018, a study was conducted in Kuwait to investigate the public’s opinion about whether physical violence was justified against females in the name of “honor” and whether the subjects were for or against the setting of legislation allowing such violence. The study included 1050 citizens, and in terms of methodology, it was performed in a way that minimized biases as much as possible (item order randomization, use of vignettes, and surveys). The results showed that half of the sample found it acceptable to practice physical violence against females in case of adultery, and a third (of both men and women) encouraged setting legislation to punish females who commit adultery. These results could be attributed to the extremely strong involvement of tribes in such matters regarding honor in Kuwait, which is avoided by punishing the female severely to satisfy the tribe [25]. 

A recent study from Pakistan that investigated attitudes regarding HK included a sample of 695 participants aged between 18 and 55 years. The study revealed that there were no major attitude differences between the genders. Moreover, the study showed that individuals who lived in rural settings, were older adults, or were members of extended or joint families expressed higher affirmation for HK than those who resided in urban settings, were younger adults, or were members of nuclear families [26]. 

## 6. Legal Aspects of Honor Killing

Violence against women in many cultures is perceived as a way of protecting the family’s name and ending the shame that the female brings. Killing, from a legal perspective, is prohibited and punished to the extremes. This is not the case for crimes in the name of honor; these types of killings are considered justified to receive lesser punishment if conceptualized to be honor crimes [27]. Many studies have attributed these cultures to be Islamic or of Middle Eastern descent, but unfortunately, honor crimes are growing into a universal pattern [27]. 

To this day, killings in the name of honor are treated to a lesser extent, and sometimes, the perpetrator is exempted from punishment. For example, in Egypt, Penal Code no. 58 1937 of Article 237 states that the perpetrator is “liable to prison sentence instead of capital punishment.” Similarly, in Iraq, the perpetrator is only “punished by prison for a period not exceeding three years,” as stated by Article 409 Penal Code 1966. In addition, United Arab Emirates Article 334 of law no. 3/1978 states that the “perpetrator shall be punished with a prison sentence” [27]. 

In Kuwait, according to Article 153 of the Penal Code, the perpetrator is punished by “prison for a period not more than 3 years and a fine or by one of these two penalties”. In Jordan, the perpetrator may benefit from a mitigating excuse, which means that the act of killing in the name of protecting the family’s honor is justifiable and punished to a lesser extent [27]. Studies conducted in Pakistan have concluded that in relation to legal action toward HK, only a small number of perpetrators were arrested. In addition, governmental and local authorities avoided these types of cases, as in they looked the other way, contributing to the support of these violent crimes. On the other hand, in countries where HKs are considered illegal, lawyers are often found to use the “defense in the name of honor”, in which a woman is considered the property of men, and the act of killing is self-defense. Other lawyers may use the defense of “temporary insanity” to exempt the perpetrators from any punishment they might face if ever caught [2]. 

Similar legal actions were observed in Palestine, such as the silence culture and the cover up of HKs. Many crimes were under-reported, and for whatever cases that did reach the judicial system, certain patterns of silence were noticed. For example, soon after reporting, witnesses would disappear, never to be seen again. In addition, some witnesses with further investigations would change their statements, contradicting any past statements. Furthermore, scenes of the crime were often distorted, and no viable evidence was able to be found. Finally, people would adopt internal solutions between them, and these cases were hushed with money and never asked about again [10]. 

Furthermore, in the Iran Penal Code, Article 301 justifies a father or paternal grandfather killing his daughter without any retaliation from the judicial system. In addition, Article 630, allows a man to kill his wife if he witnesses adultery [14].

## 7. Limitations of the Review

There are some possible limitations of this review. As this is a narrative review article, some recent published articles discussing honor killing may not be covered in this review. A systematic review covering various databases with more sociological and cultural focus would have been a better option for a comprehensive review.

## 8. Conclusions 

In conclusion, despite the under-reporting of HKs, it remains prevalent, leading to many innocent lives being lost under false pretenses. Honor crimes are a major issue that needs to be addressed firmly, especially in areas of poor socio-economic status and in countries exhibiting a patriarchal hierarchy. This review clearly emphasizes how crucial this topic is and how a serious urgent intervention in terms of legislation is needed, as these crimes are often given justification, and the laws of many EMR countries can be permissive regarding this issue. The lack of strict laws has affected females’ sense of safety and security and added to the patriarchal hierarchy. Additionally, more research needs to be conducted on the topic of HK to fill the gaps in literature. Furthermore, a greater willingness is required to change the unjust patriarchal infrastructure of many societies in the Eastern Mediterranean Region and to put an end to the patriarchal dominance practiced against women, as in such societies, those crimes are argued to hold a somewhat subjective moral justification.

## Data Availability

Not applicable.
